# Prognostic Nomogram Based on Circular RNA-Associated Competing Endogenous RNA Network for Patients with Lung Adenocarcinoma

**DOI:** 10.1155/2021/9978206

**Published:** 2021-08-28

**Authors:** Yang Li, Rongrong Sun, Rui Li, Yonggang Chen, He Du

**Affiliations:** ^1^Department of Central Laboratory, Affiliated Xuzhou Central Hospital, Clinical School of Xuzhou Medical University, Xuzhou 221009, China; ^2^Department of Medical Oncology, Affiliated Xuzhou Central Hospital, Clinical School of Xuzhou Medical University, Xuzhou 221009, China; ^3^Department of Clinical Pharmacy, Xuzhou Central Hospital, Clinical School of Xuzhou Medical University, Xuzhou 221009, China; ^4^Department of Medical Oncology, Affiliated Shanghai Pulmonary Hospital, Tongji University, Shanghai 200433, China

## Abstract

Evidence is increasingly indicating that circular RNAs (circRNAs) are closely involved in tumorigenesis and cancer progression. However, the function and application of circRNAs in lung adenocarcinoma (LUAD) are still unknown. In this study, we constructed a circRNA-associated competitive endogenous RNA (ceRNA) network to investigate the regulatory mechanism of LUAD procession and further constructed a prognostic signature to predict overall survival for LUAD patients. Differentially expressed circRNAs (DEcircRNAs), differentially expressed miRNAs (DEmiRNAs), and differentially expressed mRNAs (DEmRNAs) were selected to construct the ceRNA network. Based on the TargetScan prediction tool and Pearson correlation coefficient, we constructed a circRNA-associated ceRNA network including 11 DEcircRNAs, 8 DEmiRNAs, and 49 DEmRNAs. GO and KEGG enrichment indicated that the ceRNA network might be involved in the regulation of GTPase activity and endothelial cell differentiation. After removing the discrete points, a PPI network containing 12 DEmRNAs was constructed. Univariate Cox regression analysis showed that three DEmRNAs were significantly associated with overall survival. Therefore, we constructed a three-gene prognostic signature for LUAD patients using the LASSO method in the TCGA-LUAD training cohort. By applying the signature, patients could be categorized into the high-risk or low-risk subgroups with significant survival differences (HR: 1.62, 95% CI: 1.12-2.35, log-rank *p* = 0.009). The prognostic performance was confirmed in an independent GEO cohort (GSE42127, HR: 2.59, 95% CI: 1.32-5.10, log-rank *p* = 0.004). Multivariate Cox regression analysis proved that the three-gene signature was an independent prognostic factor. Combining the three-gene signature with clinical characters, a nomogram was constructed. The primary and external verification *C*-indexes were 0.717 and 0.716, respectively. The calibration curves for the probability of 3- and 5-year OS showed significant agreement between nomogram predictions and actual observations. Our findings provided a deeper understanding of the circRNA-associated ceRNA regulatory mechanism in LUAD pathogenesis and further constructed a useful prognostic signature to guide personalized treatment of LUAD patients.

## 1. Introduction

Lung cancer is the leading cause of malignancy-related death worldwide. Lung adenocarcinoma (LUAD) accounts for ~50% of all types of lung cancer and has been on the rise year after year, especially in women and young adults. Despite recent advances in diagnostic and therapeutic approaches, the 5-year survival rate for LUAD is <20% [1]. In addition, the underlying molecular mechanisms of LUAD still remain unclear. Therefore, identifying the key molecular mechanisms of LUAD and establishing effective prognostic models for individualized treatment are urgent.

As the boom of sequencing technology and bioinformatics methods, increasing noncoding RNAs (ncRNAs) were identified, one of which is circular RNA (circRNA). circRNA, featuring a stable structure and high tissue-specific expression widely expressed, has a covalently closed loop structure in which the 3′ and 5′ ends are linked in a noncollinear way by a process termed “back-splicing” [2–4]. In recent years, thousands of circRNAs have been identified in various organisms and found to be closely associated with many diseases, particularly with cancer [5–7]. For example, Tian et al. reported that has_circ_0003159 expression was significantly downregulated in gastric cancer and associated with lymphatic node metastasis and distal metastasis [8]. Gao et al. showed that has_circ_0018289 was upregulated in cervical cancer and promotes proliferation, migration, and invasion of tumor cells [9]. Such evidences strongly support that circRNAs play critical roles in tumor progression. Hence, exploring the regulatory mechanism of circRNAs in tumors is necessary and may be beneficial for tumor therapy.

There is increasing evidence that circRNA contains microRNA response elements (MREs) and inhibits the function of microRNA (miRNA) as miRNA sponges through the competing endogenous RNA (ceRNA) network [10]. The ceRNAs are implicated in many biological processes, and the equilibrium of ceRNAs and miRNAs can be critical for the promotion of diseases. For example, Sang et al. reported that hsa_circ_0025202 could act as a miRNA sponge for miR-182-5p and further regulate the expression and activity of FOXO3, regulating tamoxifen sensitivity and tumor progression in breast cancer [11]. Fang et al. showed that circRNA-100290 could promote colorectal cancer progression through miR-516b-induced downregulation of FZD4 expression and Wnt/*β*-catenin signaling [12]. In addition, Chen et al. found that has_circ_100395 regulates the miR-1228/TCF21 pathway to inhibit lung cancer progression [13]. Collectively, these findings show that dysregulation of important circRNAs in the ceRNA network disrupts the miRNA-mediated circRNA/mRNA interactions and therefore contributes to cancer initiation and progression. However, systematic analysis of the circRNA-associated ceRNA network in LUAD remains insufficient and requires further exploration.

In this study, based on the public cohorts downloaded from Gene Expression Omnibus (GEO) and The Cancer Genome Atlas (TCGA), we aimed to detect the aberrant key circRNAs and explore the potential regulatory mechanism by constructing a circRNA-associated ceRNA network for LUAD patients. Using the LASSO method, we then established a mRNA-based prognostic signature derived from the ceRNA network and validated the prognostic performance in an independent cohort. Besides, combining the prognostic signature with clinical characters, we constructed a clinical nomogram that could serve as a useful guide toward more effective individualized treatment decisions for LUAD patients.

## 2. Materials and Methods

### 2.1. Data Processing

Two circRNA expression profiles (GSE101586 and GSE101684) were obtained from the GEO (https://www.ncbi.nlm.nih.gov/geo/) database. GSE101586, including 5 paired tumor and adjacent normal lung samples, was generated using the Agilent-069978 Arraystar Human CircRNA microarray V1, which contains 5490 circRNA probes. GSE101684, including 4 paired tumor and adjacent normal lung samples, was generated using the Agilent-074301 Arraystar Human CircRNA microarray V2, which contains 9114 circRNA probes. These raw data were processed by background correction and normalization using the “affy” package of R/Bioconductor. The expression data of mRNA (513 LUAD tissues and 59 normal tissues) and miRNA (513 LUAD tissues and 46 normal tissues) were retrieved from TCGA (https://portal.gdc.cancer.gov/). The normalized count values of level 3 gene expression data derived from Illumina HiSeqV2 were extracted as gene expression measurements. Clinical information of 513 LUAD patients was obtained. Forty-seven LUAD patients were excluded because of unknown survival time, age, and tumor stage. Ultimately, 466 patients were retained in our study. An independent cohort (GSE42127) collected from GEO contained 133 LUAD patients, which was used to test the prognostic ability as an independent data set. The Robust Multi-array Average algorithm was used for preprocessing the raw data [14]. Clinical information is described in Table 1.

### 2.2. Selection of Differentially Expressed RNAs

Differentially expressed circRNAs (DEcircRNAs) were identified by the Student *t*-test with *p* < 0.05 between LUAD and adjacent normal lung tissues. DEcircRNAs overlapped between GSE101586 and GSE101684 were selected to construct the ceRNA network. The edgeR package was used to identify the differentially expressed miRNAs (DEmiRNAs) and differentially expressed mRNAs (DEmRNAs). The cutoff values were set at the FDR < 0.05 and ∣log2FC | >2.

### 2.3. Constructing the circRNA-Associated ceRNA Network

A circRNA-associated ceRNA network was constructed based on the interactions between DEcircRNAs, DEmiRNAs, and DEmRNAs. The TargetScan prediction tool was used to identify interactions between DEcircRNAs with the target miRNAs. To obtain high-quality circRNAs acting as miRNA targets and distinguish those circRNAs acting as miRNA decoys, the circRNAs that had perfect nucleotide pairing between the 2nd and 8th positions of the 5′ end of miRNA sequences were selected [15]. The miRNAs within circRNA-miRNA interactions were further screened by DEmiRNAs identified from TCGA. The remained DEmiRNAs were further used to screen the interactions between miRNA and mRNA predicted by TargetScan. The mRNAs targeted by DEmiRNAs were intersected with the DEmRNAs identified from TCGA. The correlation between DEmiRNAs and DEmRNAs was further calculated by the Pearson correlation coefficient. Only the interactions with significant negative correlations were retained. Finally, removing the nodes that could not form a circRNA-miRNA-mRNA axis, a circRNA-associated ceRNA network was established and further visualized by Cytoscape software (version 3.7.0; http://www.cytoscape.org).

### 2.4. Functional Enrichment Analysis

In order to explore the biological functions in which the circRNA-associated ceRNA network might be involved, we selected the DEmRNAs within the ceRNA network to make the functional enrichment analysis using the Database for Annotation, Visualization, and Integrated Discovery (DAVID; http://www.david.abcc.ncifcrf.gov/). Gene Oncology involved three categories: biological processes, molecular function, and cellular components. Pathway enrichment was carried out using the Kyoto Encyclopedia of Genes and Genomes (KEGG, https://www.kegg.jp/), and it contains information about genomes, chemical substances, biological pathways, and diseases.

### 2.5. Construction of the Protein-Protein Interaction (PPI) Network

PPI information can be evaluated by an online tool, the Search Tool for the Retrieval of Interacting Genes/Proteins (STRING) database (https://string-db.org/). This database has a comprehensive score for each PPI relationship pair that is distributed between 0 and 1; the higher the score, the more reliable the PPI relationship. In this study, we applied STRING to explore the potential correlation between DEmRNAs within the ceRNA network with a medium confidence criterion (confidence score ≥ 0.4). The Cytoscape software was used to visualize the PPI network.

### 2.6. Establishment of Prognostic Signature

Based on DEmRNAs within the PPI network, univariate Cox regression analysis was utilized to test the association between gene expression and overall survival (OS) using the TCGA-LUAD training cohort. Genes with significant association were selected to construct the prognostic signature by the LASSO method using the R package “glmnet” in the TCGA-LUAD training cohort. The basic idea of LASSO is to select the variables of the sample data under the constraint that the sum of the absolute values of the regression coefficients is less than a constant, so as to minimize the sum of the squares of the residuals and make some regression coefficients strictly equal to 0. To achieve the purpose of feature selection and obtain an optimal model subsequently, the variable with a coefficient equal to 0 is regarded as a nonsignificant variable and is directly discarded. The model was applied to the expression matrix of candidate genes, and the optimal value of the penalty parameter *λ* was selected to calculate the coefficient of each gene constituting a prognostic signature. Using the combination of weighted gene expression values, a risk scoring model was established and the risk scores were calculated as shown in the following equation:

(1) $$\begin{array}{c} \text{Risk score}=\overset{n}{\underset{i=1}{\sum }}\text{E}\text{xp}i\beta i. \end{array}$$

Exp*i* represents the expression of Gene *i*. *βi* is the regression coefficient of Gene *i*, which represents the contribution of Gene *i* to the prognostic risk score. Using the median risk score as the cutoff point, patients in each cohort were divided into the low-risk or high-risk group correspondingly.

### 2.7. Statistical Analysis

A *t*-test was used to observe the gene expression difference between metastasis samples and nonmetastasis patients. The multivariate Cox proportional hazards regression model was used to evaluate independent association between prognostic signature and patient survival after adjusting for stage, age, and gender. Hazard ratios (HRs) and 95% confidence intervals (CIs) were computed based on the Cox regression analysis. Survival curves were estimated using the Kaplan-Meier method and were compared using the log-rank test. Fisher's exact test was used to observe the differences in the mortality rate and lymph node metastasis rate between different risk groups. Clinical risk factors were identified using multivariate Cox proportional hazards regression, based on which we constructed a prognostic nomogram for predicting the 3- and 5-year OS in the TCGA-LUAD cohort. The discriminatory ability of the nomogram was evaluated by calculating the concordance index (*C*-index) to measure the variation between the predicted and observed outcomes. Calibration plots were used to compare the observed and predicted probabilities for the nomogram. The significance was defined as a *p* value of <0.05. All statistical analysis was performed using R3.4.0.

## 3. Results

### 3.1. Identification of Differentially Expressed RNAs

**Figure 1:**
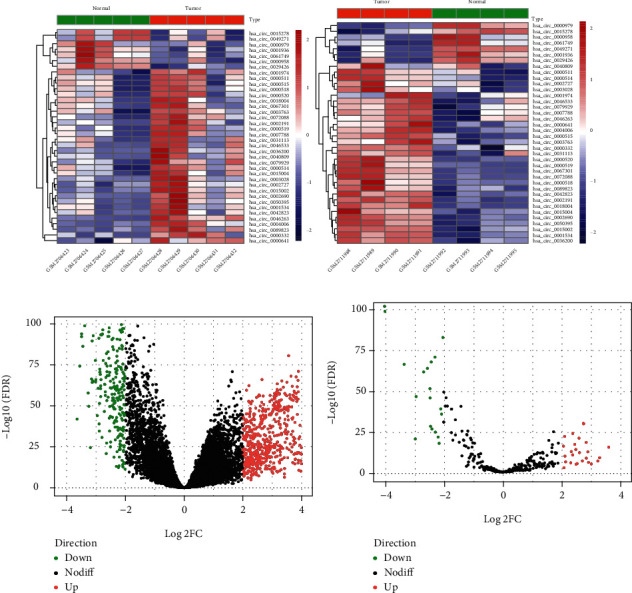
Heat maps of differentially expressed circular RNAs (a) and volcano plots of differentially expressed mRNA (b) and differentially expressed miRNA (c). Red and green dots represent significantly upregulated and downregulated RNAs, respectively (FDR < 0.05 and ∣log2FC | >2.0).

We initially performed differential expression analysis by comparing circRNA expression between LUAD and adjacent normal lung tissues in the two cohorts from GEO. With cutoff criteria of *p* < 0.05, a total of 38 DEcircRNAs (including 31 upregulated circRNAs and 7 downregulated circRNAs) were in concordance between GSE101586 and GSE101684 (Figure 1(a)). The expression data of miRNAs and mRNAs downloaded from TCGA were analyzed using the “edgeR” package in R. With cutoff criteria of FDR < 0.05 and ∣log2FC | >2.0, 56 differentially expressed miRNAs (including 37 upregulated miRNAs and 19 downregulated miRNAs) and 960 differentially expressed mRNAs (including 653 upregulated mRNAs and 307 downregulated mRNAs) were identified, respectively (Figures 1(b) and 1(c)).

### 3.2. Construction of the circRNA-Associated ceRNA Network

**Figure 2:**
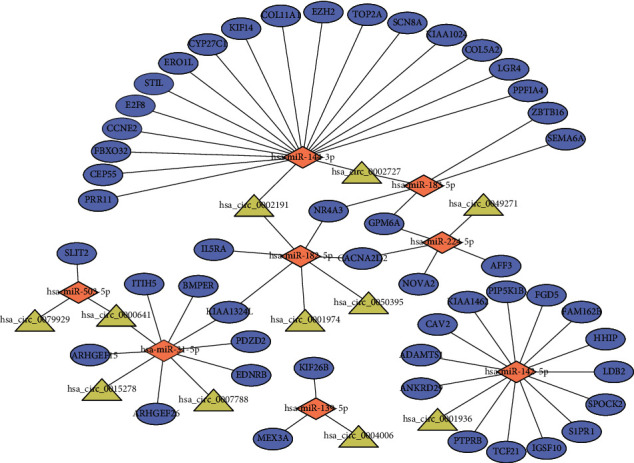
The circRNA-associated competing endogenous network in lung adenocarcinoma. Triangles represent circRNAs, diamonds represent miRNAs, ellipses represent mRNAs, and black lines represent circRNA-miRNA-mRNA interactions.

Using the TargetScan prediction tool to identify DEcircRNAs with the target miRNAs, we identified 494 circRNA-miRNA pairs based on 38 DEcircRNA and 221 miRNAs. After screening by the DEmiRNAs identified from the TCGA-LUAD cohort, only 39 circRNA-miRNA pairs remained, including 18 DEcircRNAs and 18 DEmiRNAs. We further identified mRNAs targeted by these DEmiRNAs from the TargetScan database and selected those that were overlapped with the DEmRNAs identified from the TCGA-LUAD cohort. A total of 305 miRNA-mRNA pairs were identified, including 11 DEmiRNAs and 191 DEmRNAs. After calculating the expression correlation between miRNA and mRNA by the Pearson correlation coefficient, only 53 miRNA-mRNA pairs with significant correlation were retained. Finally, combining the circRNA-miRNA pairs with miRNA-mRNA pairs, a circRNA-miRNA-mRNA network was constructed, including 11 DEcircRNAs, 8 DEmiRNAs, and 49 DEmRNAs, and subsequently visualized by Cytoscape (Figure 2).

### 3.3. Functional Enrichment

**Figure 3:**
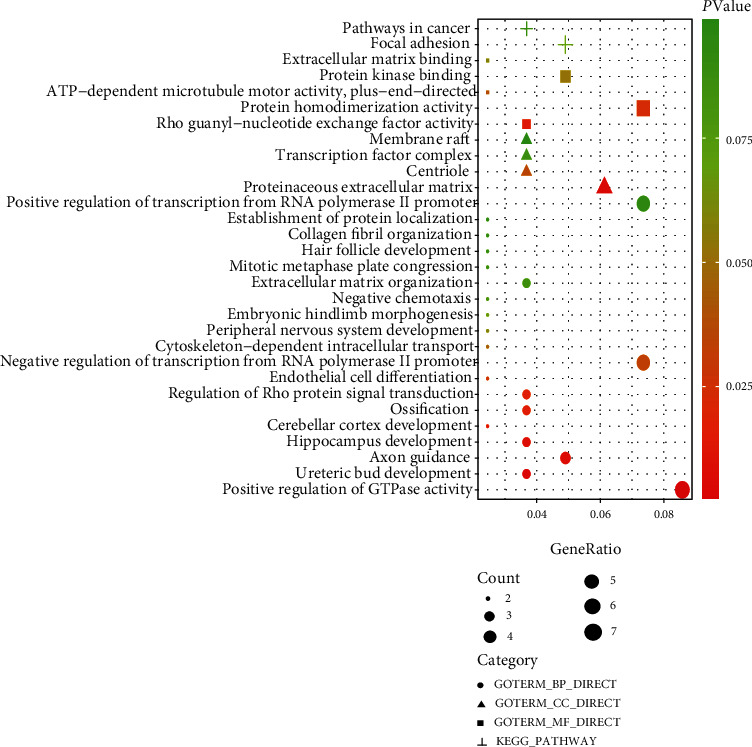
Functional enrichment of differentially expressed mRNAs in the competing endogenous RNA network.

To further investigate the biological processes in which the circRNA-associated ceRNA network might be involved, we performed GO annotation and KEGG pathway enrichment using the DAVID database. Functional enrichment results are shown in Figure 3. For GO annotation, ten GO terms were significantly enriched. Most terms, such as positive regulation of GTPase activity, endothelial cell differentiation, and regulation of transcription from RNA polymerase II promotion, had been reported in multiple articles related to the progression and metastasis of cancer. KEGG pathway enrichment analysis indicated that two pathways, including pathways in cancer and focal adhesion, were significantly enriched. Such results showed that DEmRNAs within the network played key roles in multiple cancer-related functional pathways and further indicated that the circRNA-associated ceRNA network might be involved in LUAD carcinogenesis and progression.

### 3.4. Construction of the PPI Network and Evaluation of Relationship with Clinical Characteristics

**Figure 4:**
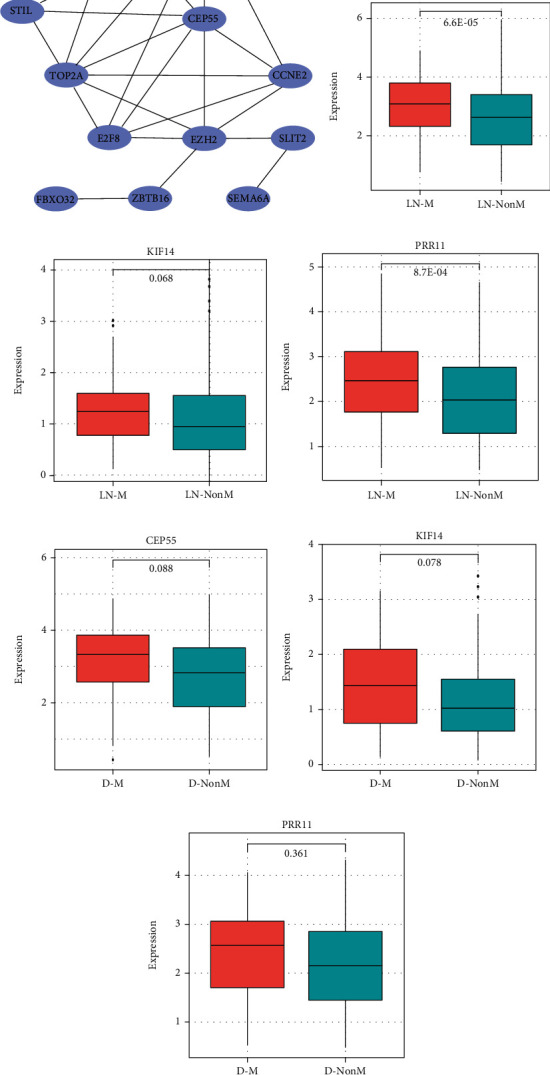
Construction of protein-protein interaction network and expression difference analysis of three genes: (a) protein-protein interaction network; (b–d) expression difference of CEP55, KIF14, and PRR11 between lymphatic node metastasis (LN-M) and nonmetastasis (LN-NonM) samples, respectively; (e–g) expression difference of CEP55, KIF14, and PRR11 between distant metastasis (D-M) and nonmetastasis (D-NonM) samples, respectively.

To explore the interactions between the 49 DEmRNAs, we constructed a PPI network using the STRING database. After removing unconnected nodes, the PPI network comprised 12 nodes and 27 edges (Figure 4(a)). Furthermore, we investigated the correlation between the gene expression and clinical variables including lymphatic node metastasis, distant metastasis, and overall survival. Univariate Cox regression analysis results showed that the high expression of three genes (including CEP55, KIF14, and PRR11) predicted a poor prognosis in LUAD patients (Table 2). Notably, we analyzed the expression difference between different lymphatic node metastasis status and distant metastasis status, respectively (Figures 4(b)–4(g)). The results showed that CEP55 and PRR11 were significantly upregulated in patients with lymphatic node metastasis. The expression of KIF14 also had an upward trend in lymphatic node metastasis patients. Although no statistical significance was observed, the average expression levels of all three genes increased in distant metastasis patients. These results indicated that these three genes might play key roles in the progression and prognosis of LUAD.

### 3.5. Construction and Validation of the Prognostic Signature

**Figure 5:**
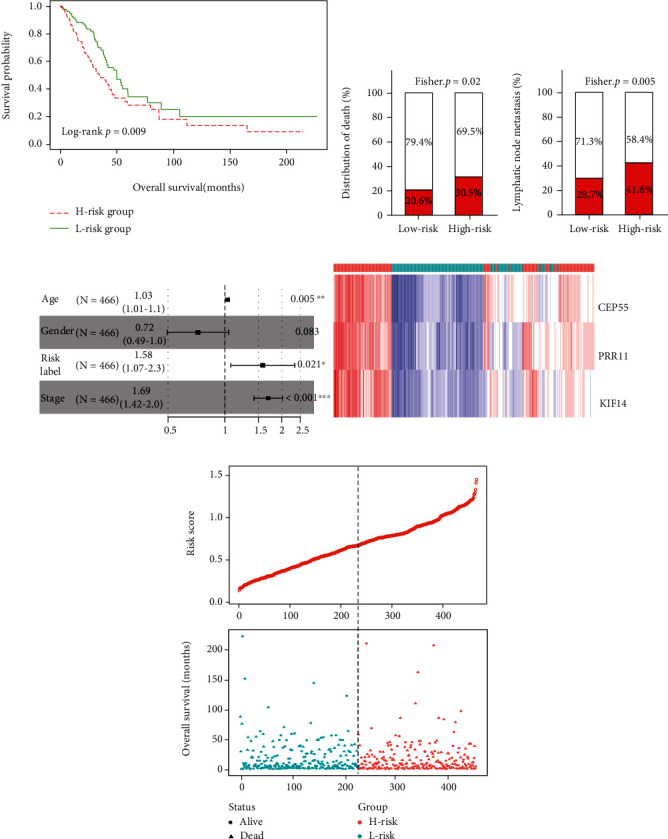
Construction of the three-gene prognostic signature: (a) Kaplan-Meier curve of the overall survival for high-risk and low-risk scores ranking by the three-gene prognostic signature; (b) the distribution of death in the high-risk and low-risk groups; (c) the distribution of lymphatic node metastasis in the high-risk and low-risk groups; (d) risk score distribution, survival status, and expression heat map of three genes corresponding to each sample above.

**Figure 6:**
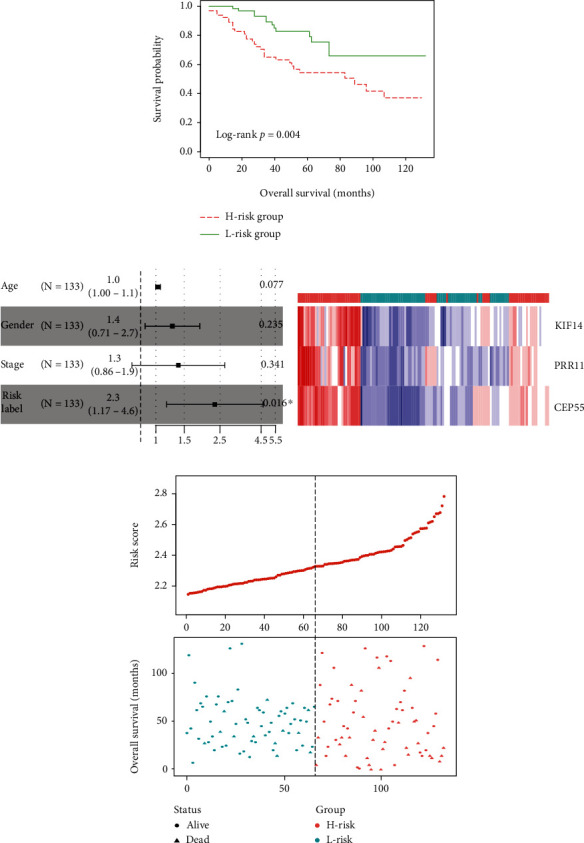
Validation of the three-gene prognostic signature in an independent cohort: (a) Kaplan-Meier curve of the overall survival for high-risk and low-risk scores ranking by the three-gene prognostic signature; (b) risk score distribution and survival status for each patient; (c) expression heat map of three genes corresponding to each sample above.

Based on the importance of three genes in the progression and prognosis of LUAD, we tried to construct a prognostic signature for LUAD patients using the LASSO method. Combining the regression coefficients with gene expression values, the risk score formula was created as follows: risk score = (0.058∗expression level of CEP55) + (0.065∗expression level of KIF14) + (0.202∗expression level of PRR11). We then calculated the risk score for each patient and ranked them based on an increasing score, after which patients were classified into a high-risk (*n* = 233) or a low-risk (*n* = 233) group based on the median risk score. We observed the overall survival between two risk groups with significantly different survival rates (log-rank *p* = 0.009; Figure 5(a)). Patients with a high-risk score had significantly shorter OS than patients with a low-risk score. The mortality rate was 30.5% (71/233) in the high-risk group, as compared to 20.6% (48/233) in the low-risk group (*p* = 0.021, Fisher exact test, Figure 5(b)). The rate of lymphatic node metastasis in the high-risk group was significantly higher than that in the low-risk group (*p* = 0.005, Fisher exact test, Figure 5(c)). The risk score distribution, survival status, and expression profile of the three prognostic genes are shown in Figure 5(e). Taking into account the patients' clinical features, including age, gender, and stage, the multivariate Cox regression analysis showed that the three-gene signature also had statistical significance as an independent prognostic factor in the training cohort (HR: 1.58, 95% CI: 1.07-2.3, *p* = 0.021, Figure 5(d)). We test the prognostic performance of the three-gene signature in an independent cohort. Similar to the training cohort findings, patients in the high-risk group had a shorter survival time than patients in the low-risk group (log-rank *p* = 0.004, Figure 6(a)). The risk score distribution, survival status, and expression profile of the three prognostic genes are shown in Figure 6(c). Multivariate Cox regression analysis showed that, after adjusting for age, gender, and stage, the prognostic signature remained significantly associated with patient OS (HR: 2.3, 95% CI: 1.17-4.6, *p* = 0.016, Figure 6(b)).

### 3.6. Development and Validation of the Nomogram Based on Three-Gene Signature

**Figure 7:**
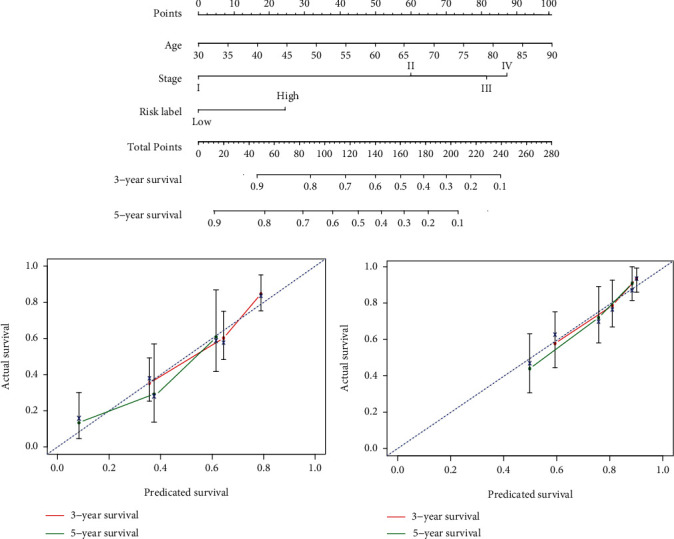
Survival nomograms (a) and calibration plots of 3- and 5-year overall survival predictions for patients with lung adenocarcinoma in the TCGA-LUAD cohort (b) and GSE42127 cohort (c).

Multivariate Cox proportional hazards regression analysis indicated that two clinical variables, including age and stage, were independent risk factors for OS. Combining clinical risk factors and the three-gene signature, we constructed a nomogram that is able to predict 3- and 5-year OS based on the multivariate Cox proportional hazards regression analysis (Figure 7(a)). The primary and external verification *C*-indexes were 0.717 and 0.716 in terms of predicting OS, respectively. The calibration plots for the probability of survival at 3 and 5 years showed good agreement between the predicted OS by nomogram and actual OS of LUAD patients in TCGA-LUAD and GSE42127, respectively (Figures 7(b) and 7(c)).

## 4. Discussion

LUAD is a major lung cancer that is in a locally advanced or metastatic stage at the time of diagnosis, which leaves no time for early detection or treatment [16]. The accurate diagnosis and prognosis may warrant timely treatment to potentially decrease mortality. Therefore, it is essential to explore the molecular mechanisms of LUAD progression and identify the effective biomarkers contributing to better treatment and better overall prognosis for LUAD patients.

Growing experimental evidences have shown that circRNAs play important roles in many complicated human diseases, including malignant tumors [17–19]. Lately, lots of studies have found that circRNAs could function as a tumor regulator in lung cancer [20–22]. As a type of high-efficiency ceRNA, it could inhibit the binding of miRNAs to target genes and regulate the expression level of target genes by exerting a miRNA sequestering effect [23]. However, the expression pattern and biological function of circRNAs in LUAD remain largely elusive. In the present study, we constructed a circRNA-miRNA-mRNA ceRNA network to explore the regulatory mechanism of circRNAs involved in tumor progression. An integrated circRNA-associated ceRNA network, including 11 circRNAs, 8 miRNAs, and 49 mRNAs, was established. The 11 circRNAs identified in the ceRNA network were hsa_circ_0002191, hsa_circ_0002727, hsa_circ_0049271, hsa_circ_0050395, hsa_circ_0001974, hsa_circ_0004006, hsa_circ_0000641, hsa_circ_0079929, hsa_circ_0007788, hsa_circ_0001936, and hsa_circ_0015278. We found that two of eleven circRNAs had been reported to be related to disease progression and pathogenesis. Peng et al. reported that hsa_circ_0015278 was significantly downregulated in papillary thyroid carcinoma, showing interactive potential with two cancer-related miRNAs [24]. Furthermore, several promising cancer-related genes that may be targets of the dysregulated hsa_circRNA_0015278/miR-141-3p/miR-200a-3p axis were identified to explore the pathogenesis of papillary thyroid carcinoma. Another circRNA, named hsa_circ_0079929, has been reported by Zou et al. [25]. They found that elevated expression of hsa_circRNA_0079929 might inhibit the expression of hsa-miR-26a-3p to increase aortic smooth muscle cell phenotype or apoptosis in thoracic aortic dissection. Although other circRNAs have not been reported previously, we could detect the interactive potential with miRNAs and target genes based on the constructed ceRNA network. For example, in the circRNA-associated ceRNA network constructed in the present study, hsa_circ_0001936 interacted with has-miR-142-5p and further might regulate its target genes, such as CAV2, FGD5, and S1PR1, which were involved in the positive regulation of the GTPase activity process. Such results indicated that hsa_circ_0001936 might play key roles in LUAD progression by regulating GTPase activity. Thus, the regulatory mechanism of these circRNAs speculated in the ceRNA network requires further experimental investigation in future studies.

Limited by the small sample size or lack of corresponding clinical data, it is not feasible to construct diagnostic or prognostic signatures for clinical application based on circRNAs at the current stage. However, extensive transcriptome data with corresponding clinical data provide us the possibility to construct clinically available diagnostic or prognostic signatures. In the present study, we identified the key mRNA participating in the circRNA-associated ceRNA network based on the STRING database and further constructed a three-gene prognostic signature for LUAD patients. The three-gene prognostic signature is composed of centrosomal protein 55 (CEP55), kinesin family member 14 (KIF14), and proline rich 11 (PRR11). CEP55 localizes to the centrosome of interphase cells and to the midbody during cytokinesis [26]. Many studies have demonstrated that CEP55 was highly expressed in a variety of cancers and could be used as a diagnostic and prognostic marker for several cancers [27–29]. For LUAD, Fu et al. had reported that CEP55 was significantly upregulated in LUAD patients and could be an independent prognostic factor [30]. As a mitotic kinesin, KIF14 has been reported to serve oncogenic roles through the regulation of the cell cycle, DNA replication, and DNA repair biological processes in a variety of malignancies, such as colorectal cancer [31], ovarian cancer [32], gastric cancer [33], and prostate cancer [34]. Zhang et al. had confirmed that KIF14 was notably upregulated in tumor tissues of LUAD, and the expression levels of the KIF14 exhibited a strong correlation with OS [35]. PRR11, located on chromosome 17q22, has been reported to be closely associated with cell cycle progression and was also demonstrated to participate in various biological processes in tumor cells, including cell invasion, migration, and proliferation, by acting as an oncogene [36–39]. Ji et al. found that PRR11 was periodically expressed in a cell cycle-dependent manner. RNAi-mediated silencing of PRR11 caused S phase arrest and suppressed cellular proliferation and colony formation ability in lung cancer cells, demonstrating that PRR11 had a critical role in both cell cycle progression and tumorigenesis [40]. All three genes are associated with tumor progression and prognosis, indicating that the three-gene signature could be served as a clinically available prognostic signature to provide potential novel targets and promote individualized treatment. In addition, we found that all three genes were regulated by has-miR-144-3p interacting with hsa_circ_0002191 and hsa_circ_0002727, indicating that hsa_circ_0002191 and hsa_circ_0002727 might serve as potential regulators to affect gene expression and further influenced the progression and prognosis of LUAD patients. Thus, further experimental investigation needs to be implemented to explore the regulatory mechanism and prognostic ability of hsa_circ_0002191 and hsa_circ_0002727.

## 5. Conclusions

The present study constructed and analyzed a circRNA-associated ceRNA network via comprehensive bioinformatics analysis, which could provide meaningful evidences for future studies focused on the molecular mechanisms of LUAD [41]. Based on the circRNA-associated ceRNA network, a three-gene prognostic signature was constructed. Furthermore, combining the prognostic signature with clinical risk factors, we constructed a clinical nomogram for predicting 3- and 5-year OS that could serve as a useful guide toward more effective individualized treatment decisions for LUAD patients.

## Figures and Tables

**Table 1:** Clinical information analyzed in this study.

	TCGA-LUAD	GSE42127
Sample		
Normal	—	—
Tumor	466	133
Mean age (years; range)	65 (33-88)	66 (42-86)
Gender		
Male	213	68
Female	253	65
Stage		
I	254	89
II	110	22
III	77	20
IV	25	2
Status		
Alive	347	90
Dead	119	43
Platform	Illumina HiSeqV2	Illumina Human WG-6 v3.0

**Table 2:** Univariate Cox analysis of DEmRNAs within protein-protein interaction network.

DEmRNAs	HR	95% CI:low	95% CI:high	*p* value	DEdir
TOP2A	1.006	0.998	1.014	0.161	Up
CEP55	1.037	1.012	1.063	0.004	Up
E2F8	1.058	0.977	1.146	0.166	Up
CCNE2	1.062	0.945	1.194	0.310	Up
KIF14	1.128	1.038	1.226	0.004	Up
EZH2	1.003	0.958	1.049	0.903	Up
STIL	1.063	0.965	1.171	0.215	Up
PRR11	1.085	1.041	1.130	<0.001	Up
SLIT2	1.031	0.949	1.120	0.472	Down
ZBTB16	0.994	0.876	1.127	0.921	Down
FBXO32	1.012	0.995	1.030	0.171	Up
SEMA6A	1.109	0.939	1.310	0.223	Down

Notes: DEmRNAs: differentially expressed mRNAs; HR: hazard ratio; CI: confidence interval; DEdir: differentially expressed direction.

## Data Availability

The source data of this study were derived from the public repositories, as indicated in Materials and Methods. And all data that support the findings of this study are available from the corresponding author upon reasonable request.

## Abbreviations

LUAD: Lung adenocarcinoma
ncRNAs: Noncoding RNAs
circRNA: Circular RNA
MREs: MicroRNA response elements
ceRNA: Competing endogenous RNA
GEO: Gene Expression Omnibus
TCGA: The Cancer Genome Atlas
DAVID: Database for Annotation, Visualization, and Integrated Discovery
GO: Gene Oncology
KEGG: Kyoto Encyclopedia of Genes and Genomes
STRING: Search Tool for the Retrieval of Interacting Genes/Proteins
PPI: Protein-protein interaction
LASSO: Least absolute shrinkage and selection operator
OS: Overall survival
CI: Confidence interval
HR: Hazard ratios
*C*-index: Concordance index.
